# Finding smORFs: getting closer

**DOI:** 10.1186/s13059-015-0765-3

**Published:** 2015-09-14

**Authors:** Juan Pablo Couso

**Affiliations:** School of Life Sciences, University of Sussex, Falmer, Brighton BN1 6PU UK

## Abstract

Millions of small open reading frames exist in eukaryotes. We do not know how many, or which are translated, but bioinformatics is getting us closer to the answer.

See related Research article: http://www.genomebiology.com/2015/16/1/179

DNA sequences encoding small open reading frames (smORFs) of fewer than 100 amino acids (aa) exist in each eukaryotic genome in numbers several orders of magnitude higher than the corresponding annotated protein-coding genes (Fig. 1). Due to difficulties with bioinformatic detection and experimental analysis, along with their sheer numbers, smORFs have been ignored for a long time by mainstream genomics. Thanks to recent advances in bioinformatic and experimental techniques, however, smORFs are receiving increasing attention. Extensive use of RNA-Seq has shown that thousands of smORFs are transcribed, in many cases, in putative noncoding RNAs, and high-throughput experimental techniques have detected translation of a few hundred of these. However, the possibility remains that many more smORFs are functional, but yet uncharacterized. Bioinformatic methods followed by targeted experimental verification are needed to improve the identification of putative functional smORFs. A new paper in *Genome Biology* [1] provides a significant step towards such a solution.

**Fig. 1:**
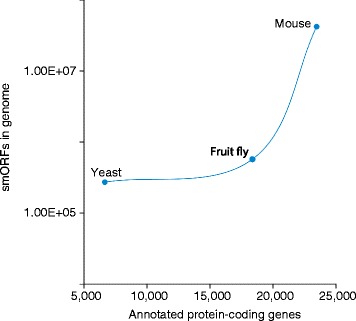
The number of small open reading frames (smORFs) in eukaryotic genomes (shown in log scale) greatly exceeds that of annotated protein-coding genes, and reaches 265,000 in yeast [4], 556,000 in the fruit fly *Drosophila* [2], and 40,700,000 in mouse [3]. Note that the current number of corroborated functional smORFs is but a small fraction of these (see text and [1] for details). The number of annotated protein-coding genes was obtained from the Saccharomyces Genome Database (yeast; http://www.yeastgenome.org/), FlyBase (fruit fly; http://flybase.org/), and Ensembl (mouse; http://www.ensembl.org/index.html) (accessed 12 August 2015)

## The trouble with smORFs

smORFs without experimental evidence of function or homology with other protein-coding genes are simply discarded by genome annotations. This arbitrary cut-off makes sense for three main reasons. First, smORFs present a problem because of their high number. Classical, presequencing genetics data can be viewed as consistent with the thousands of annotated genes in sequenced animals, but they are at odds with the notion that protein-coding genes are in the hundreds of thousands in yeast and fruit flies, and in the millions in mammals (Fig. 1).

Second, standard computational methods for gene discovery do not work well with smORFs. The accepted way to verify whether an open reading frame (ORF) is actually a gene-coding sequence is by detecting either its similarity to previously identified proteins or its conservation across related species. With access to gene sequences, this approach seems to be computationally trivial. However, Blast and related methods are size-dependent (as they measure absolute amount of conservation, i.e., the number of conserved positions), and short sequences are physically unable to obtain the high conservation scores that are the accepted indicator of functionality for canonical proteins. This Blast “handicap” starts to have an effect below 80 aa and this becomes extreme below 20 aa [2]. Standard measures of relative conservation do not work well either. For canonical protein-coding ORFs there is conservation of amino acid sequence across different species, and the *K*_a_/*K*_s_ metric measures this purifying selection at the nucleotide level. Thus, comparisons of functional ORFs between related species are expected to display a prevalence of synonymous versus nonsynonymous codon substitutions (a ratio *K*_a_/*K*_s_ <1). However, it is difficult to score statistically significant values for very short sequences because the number of possible changes is low, such that *K*_a_/*K*_s_ loses predictive power below 100 aa [2]. Tailored approaches to smORF verification have given disparate numbers [2, 3] and have been hindered by the high number of smORF candidates. In addition, there is a lack of clear criteria for the specific features of functional smORFs for use in bioinformatics analyses, due to the absence of an adequate number of experimentally corroborated smORFs.

Third, experimental approaches have not clarified this smORF mystery in multicellular organisms. Attempts have been made to experimentally screen smORFs at a genomic level in unicellular eukaryotes such as yeast, showing that hundreds of smORFs can produce a phenotype [4] and refuting the null hypothesis that “smORFs are irrelevant and nonfunctional”. However, because of high smORF numbers, a genome-wide experimental characterization of smORF function in metazoans has not been possible, even in invertebrate models such as *Caenorhabditis elegans* and *Drosophila*. A catch-22 situation then ensues, whereby mutations and other indicators of functionality are assigned to nearby genes because smORFs are not annotated. Despite this, the biological function of a handful of metazoan smORFs has been experimentally ascertained, including features such as molecular function, wide conservation across species, and medical relevance (reviewed in [5]). However, we simply have no idea of how many other functional smORFs remain uncharacterized.

## Quality time for smORF conservation

Mackowiak et al. [1] bring a new approach to computer-based searches for functional smORFs that is likely to represent a major advance with respect to previous studies. They analyze three qualitative features of coding sequence conservation using more advanced methods and more refined datasets than previous attempts. Furthermore, they extensively identify putative functional smORFs in five systems: humans, mouse, zebrafish, *Drosophila*, and *Caenorhabditis elegans*. The authors study three features. The first is conservation of amino acid sequence by phylogenetic codon substitution frequencies (PhyloCSF). This approach is superior to classic *K*_a_/*K*_s_, as it also takes into account further types of substitutions, such as conservative (replacing an amino acid for a similar one) and non-sense (introducing a stop codon). PhyloCSF then assigns a score to each codon substitution in an alignment based on the relative frequency of that substitution in known coding and noncoding regions. PhyloCSF has been shown to score better for sequences 30–180 nucleotides long [6], and can be used thanks to the availability of many high-quality sequenced genomes of species related to the ones used in this study.

The second feature is ORF conservation, defined as conservation of in-frame start and stop codons in related species. A similar strategy had been tried before [2], but the increase in sequenced genomes means that Mackowiak et al. were able to study ORF conservation across multiple species; in addition, the availability of extensive transcriptome data for the studied animal models allows the authors to use smORFs from transcriptomes as a starting pool, vastly reducing the likelihood of false positives. The third feature is a drop in nucleotide sequence conservation outside ORFs using PhastCons [7], which utilizes precomputed alignments of genomic sequences from several related species. The accuracy of PhastCons is very high, and when used to measure absolute conservation, it can identify protein-coding sequences as short as 50 aa [8]. However, here only local relative changes in conservation are used [1]. Again, increased availability of genome sequence data from nonmodel species is important in enabling this approach. Genomes of related species are often syntenic, facilitating the definition of alignment blocks used by PhastCons.

These features are studied in a machine-learning environment, enabling weighting of each component and eliminating the use of arbitrary cut-offs. This is possible by the current availability of a pool of previously verified smORFs to use as positive controls, and a negative control set of short ORFs in classical noncoding RNAs such as pre-miRNA, rRNA, tRNA, snRNA, and snoRNA.

The combination of these three features identifies about 2000 putative functional smORFs, including some 800 in human, 350 in mouse, 200 in zebrafish and fruit flies, and 400 in nematodes, including long noncoding RNAs (lncRNAs) and many upstream ORFs (uORFs). Interestingly, these peptides show several distinctive features. First, they are much shorter (medians ranging from 11 to 49 aa) than smORFs previously annotated as functional in these models (median just above 80 aa; see also [8]). Second, they can be widely conserved, at least across vertebrates (the apparent absence of wide conservation for *Drosophila* and *C. elegans* smORFs is likely to be due to lack of more extensive genomic data outside their immediate families). Finally, they are different from annotated proteins in terms of amino acid usage and sequence homologies (i.e., they do not contain known protein domains).

Mackowiak et al. compare these results with previous bioinformatic screens [2, 3, 9] and show that, at least for the three criteria used, their 2000 smORFs significantly outscore previous candidate sets, but also that the overlap with the previous candidate smORFs is fairly minimal. The question arises whether some smORFs do not conform to the criteria used by the authors. The emphasis on conservation might exclude novel, nonconserved peptides [9] that could be translated and functional, and also many uORFs that act as *cis* RNA elements that regulate translation [5].

## The trouble at the bench

Recent high-throughput experimental approaches such as proteomics and ribosomal profiling [3, 8, 10] have reliably detected the translation of hundreds of smORFs, representing at most a third of the smORFs that are shown to be transcribed in these studies. Similarly, the fraction of the candidates from the Mackowiak et al. study that are corroborated by these techniques is only around 10 % [1]. However, even high-throughput experimental data are likely to provide an underestimation of functional smORFs. Detection of peptides by proteomics is biased not only by the abundance of peptides, but also by their stability, which depends on size [10]. The detection of smORF peptides is further curtailed by the inability of short amino acid sequences to generate several nonoverlapping trypsinized peptides (as required by standard proteomics validation).

The comparison of ribosomal profiling and proteomics data reveals that ribosomal profiling is three to four times more sensitive, and that proteomics only reliably detects abundantly translated peptides [8]. However, again due to size, smORFs generate fewer profiling reads than canonical proteins, and thus it is difficult to unambiguously determine their translation. The detection of apparently translated smORFs in lncRNAs generates controversy, and thus published ribosomal profiling analysis may have become too conservative. Nonetheless, the main problem with ribosomal profiling is that the published data are not nearly as extensive as RNA-Seq data, as relatively few samples have been profiled.

In summary, proteomics and ribosomal profiling can corroborate bioinformatic data, but, currently, absence of experimental evidence is no reason to discard bioinformatically well-supported smORFs. In this way the “Mackowiak smORFs” provide a valuable starting point for further experimental validation. The commendable clarity and openness of the authors in their paper when presenting data for their putative functional smORFs will help to enable future progress.

## Abbreviations

aa: Amino acids
lncRNA: Long noncoding RNA
ORF: Open reading frame
PhyloCSF: Phylogenetic codon substitution frequencies
smORF: Small open reading frame
snoRNA: Small nucleolar RNA
snRNA: Small nuclear RNA
